# The “Cosmopolitan,” Club Rates

**Published:** 1890-09

**Authors:** 


THE "COSMOPOLITAN" is acknowledged to be the best, the most high-toned and refined family magazine published. It is an educator of the highest and best order;—children and grown folks alike read it with intense interest, and with profit. In our judgment it far surpasses "Harper's"—and the illustrations, fine engravings, are vastly superior to those of any magazine we have ever seen. And yet it is published at the extremely low figure of $2.40 a year. Just how they can afford it we do not know, but we are authorized to offer it in combination with DANIEL'S TEXAS MEDICAL JOURNAL at only $1.35 a year! The two for $3.35. Or, looking at it from the other side, taking the COSMOPOLITAN at the regular price, $2.40, you get the JOURNAL, which is $2.00 a year, for 95 cents; think of it. This offer is limited, and applies only to new subscribers. Send $3.35 to this office at once, and secure both these high character publications for the year, at a little more than the price of one.

